# Evaluation and Selection of Appropriate Reference Genes for Real-Time Quantitative PCR Analysis of Gene Expression in Nile Tilapia (*Oreochromis niloticus*) during Vaccination and Infection

**DOI:** 10.3390/ijms16059998

**Published:** 2015-04-30

**Authors:** Erlong Wang, Kaiyu Wang, Defang Chen, Jun Wang, Yang He, Bo Long, Lei Yang, Qian Yang, Yi Geng, Xiaoli Huang, Ping Ouyang, Weimin Lai

**Affiliations:** 1 Department of Basic Veterinary, Sichuan Agricultural University, Wenjiang District Huimin Road No. 211, Chengdu 611130, China; E-Mails: welsicau@126.com (E.W.); wangjun1986616@gmail.com (J.W.); heyang@sicau.edu.cn (Y.H.); lbsicau@126.com (B.L.); yangleigy@163.com (L.Y.); yangqiansicau@126.com (Q.Y.); gengyisicau@126.com (Y.G.); ouyang.ping@live.cn (P.O.); nwm_mm2004@163.com (W.L.); 2 Key Laboratory of Animal Disease and Human Health of Sichuan Province, Sichuan Agricultural University, Wenjiang District Huimin Road No. 211, Chengdu 611130, China; 3 Department of Aquaculture, Sichuan Agricultural University, Wenjiang District Huimin Road No. 211, Chengdu 611130, China; E-Mails: chendf_sicau@126.com (D.C.); hxldyq@126.com (X.H.)

**Keywords:** real time quantitative PCR, reference gene, stability, normalization, tilapia, rSip, *Streptococcus agalactiae*, *IgM*

## Abstract

qPCR as a powerful and attractive methodology has been widely applied to aquaculture researches for gene expression analyses. However, the suitable reference selection is critical for normalizing target genes expression in qPCR. In the present study, six commonly used endogenous controls were selected as candidate reference genes to evaluate and analyze their expression levels, stabilities and normalization to immune-related gene *IgM* expression during vaccination and infection in spleen of tilapia with RefFinder and GeNorm programs. The results showed that all of these candidate reference genes exhibited transcriptional variations to some extent at different periods. Among them, *EF1A* was the most stable reference with RefFinder, followed by *18S rRNA*, *ACTB*, *UBCE*, *TUBA* and *GAPDH* respectively and the optimal number of reference genes for *IgM* normalization under different experiment sets was two with GeNorm. Meanwhile, combination the *C*q (quantification cycle) value and the recommended comprehensive ranking of reference genes, *EF1A* and *ACTB*, the two optimal reference genes, were used together as reference genes for accurate analysis of immune-related gene expression during vaccination and infection in Nile tilapia with qPCR. Moreover, the highest *IgM* expression level was at two weeks post-vaccination when normalized to *EF1A*, *18S rRNA*, *ACTB*, and *EF1A* together with *ACTB* compared to one week post-vaccination before normalizing, which was also consistent with the *IgM* antibody titers detection by ELISA.

## 1. Introduction

Real-time fluorescent quantitative PCR (qPCR) is a highly specific, highly sensitive and well reproducible methodology with high throughput, broad dynamic range and quantitative accuracy for measuring mRNA transcript and gene expression levels from a given sample [1,2]. Unlike traditional PCR, which simply looks for a band on a gel to detect the amplification products at the end of the reaction, qPCR allows amplification and detection to proceed simultaneously [3]. Recent reports with qPCR affirm the transformation of this technology from an experimental tool into the scientific mainstream [4]. The range of application of qPCR is immense, such as measuring mRNA expression levels, DNA copy number, transgene copy number and expression analysis, allelic discrimination and measuring viral titers, *etc.* [5,6,7,8]. Thus, it has become the gold standard for accurate, sensitive and rapid quantification of gene expression [9,10].

However, among the golden rules of qPCR technique [11], transcription level normalization to the ideal endogenous control gene(s) is a key step before any meaningful comparisons of target gene expression levels is made, because multiple factors such as the different amounts of initial sample, quality and integrity of template RNA, primer design, reverse-transcription efficiency and so on, can generate errors that significantly affect analysis of results [12]. The ideal endogenous control genes, which are often called reference or housekeeping genes (HKGs), should not be regulated or influenced across different experimental conditions (treatments, tissues and developmental stages). They should be expressed with minimal innate variability between samples, as well as in abundance [13,14]. Commonly used reference genes include those encoding 18S ribosomal RNA (*18S rRNA*), β-actin (*ACTB*), α-tubulin (*TUBA*), ubiquitin-conjugating enzyme (*UBCE*), glyceraldehyde-3-phosphate dehydrogenase (*GAPDH*), and elongation factor 1 α (*EF1A*) [15,16,17]. However, with the increasing application of qPCR method, the indiscriminate use of some reference genes is more questionable, since their expression levels are regulated and vary under different treatments and in different tissues and no single gene is found to show such stable expression among a variety of species [12,14,15,18,19]. Thus, arbitrary use of reference genes without prior validation can result in the misinterpretation of the qPCR data [20], and the selection of the most stable gene or set of genes under the experimental conditions is necessary and crucial to accurate profiling of gene expression [21]. RefFinder is a comprehensive tool, which integrates the currently available major computational programs including the comparative Δ*C*_t_ method [22], BestKeeper [23], NormFinder [24], and GeNorm [25] developed for evaluating and screening reference genes from extensive experimental datasets, as well as to compare and rank the tested candidate reference genes. Based on the rankings from each program, it assigns an appropriate weight to an individual gene and calculates the geometric mean of their weights for the overall final ranking [26].

Nile tilapia (*Oreochromis niloticus*), one of the most important freshwater fish species, is recommended by the Food and Agriculture Organization (FAO) as a high quality aquaculture varieties. However, *Streptococcus agalactiae* (Group B streptococcus, GBS) has seriously plagued and damaged tilapia farming in recent years, causing both morbidity and mortality [27]. Surface immunogenic protein (Sip) is a surface-exposed protein of *S. agalactiae* and found to be highly conserved and present in every serotype of *S. agalactiae* isolates [28,29], also used as a potential vaccine candidate, has been identified for subunit vaccines or DNA vaccines in recent studies [30,31,32]. Recently, qPCR has been widely applied to a growing number of aquaculture studies for gene expression analyses in the study of subunit vaccines or DNA vaccines against bacterial infection [33,34,35]. Furthermore, reports on the selection of reference genes during vaccination and infection of tilapia with qPCR are deficient.

Immunoglobulin M, which is encoded by *IgM* gene, is produced following antigen or pathogen encounter [36,37] and commonly used as an important evaluation index of the immune effectiveness of vaccines. Several studies have shown that the expression level of immune-related gene *IgM* increased significantly during vaccination with subunit/DNA vaccines and at a different period of infection with pathogenic bacteria in fish [33,34,35,38]. Therefore, using the qPCR method to evaluate and screen the optimal reference genes and analyze the immune-related genes expression changes during vaccination and infection is very important for the study of fish disease.

In this study, the expression change of *IgM* gene was investigated in parallel with six candidate reference genes including *18S rRNA*, *ACTB*, *TUBA*, *UBCE*, *GAPDH* and *EF1A* during vaccination with vaccine and infection with bacteria in spleen of Nile tilapia. We also evaluated the expression, stability and normalization of these reference genes in order to select the best reference gene for immune-related gene expression study in Nile tilapia during vaccination and infection with qPCR.

## 2. Results

### 2.1. qPCR Amplification of Candidate Reference Genes and Target Gene

**Figure 1:**
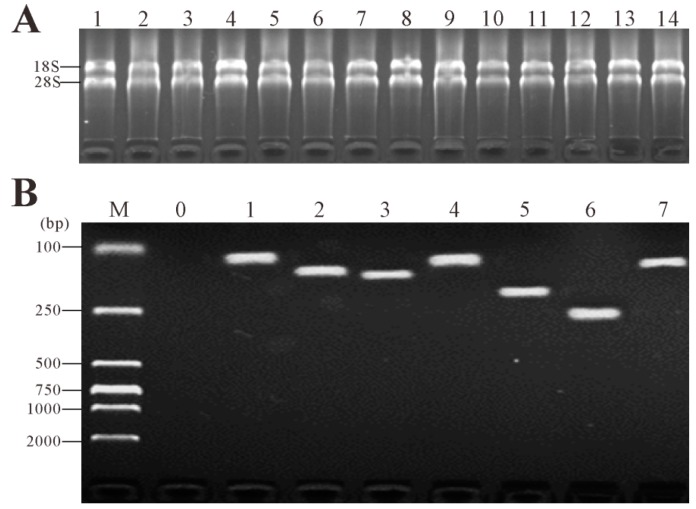
Agarose gel electrophoresis of total RNA and PCR amplification of the candidate reference genes and target gene using synthesized cDNA as templates. (**A**) Total RNA extracted from the spleen at 0w (the zero day before vaccination), 1w (1 week post-vaccination, the same below), 2w, 3w, 4w, C24h (24 h post-challenge), C2w (2 weeks post-challenge) of rSip group (**Lanes 1**–**7**) and PBS group (**Lanes 8**–**14**) were fractioned on agarose gel 2%, which showed intact 28S and 18S rRNAs were evident without any other higher molecular weight molecules; (**B**) PCR amplification of the candidate reference genes and target gene using synthesized cDNA as templates, which revealed that the specific primer for each candidate reference gene and target gene amplified a specific product consistent with the expected size on cDNA templates. M: DNA marker DL2000. **Lane 0**: negative control. **Lane 1**–**7**: *18S* *rRNA*, *ACTB*, *TUBA*, *UBCE*, *GAPDH*, *EF1A*, *IgM*, respectively.

Via gel electrophoresis, all ratios of 28S:18S rRNA were greater than 1. The ratios of OD_260_/OD_280_ were between 1.8 and 2.0 and OD_260_/OD_2__30_ ratios were all above 2. The amplification products of the candidate reference genes and target gene using synthesized cDNA as templates appeared as a single band of the expected size on 2% agarose gels (Figure 1) while RNA control (the RNA without reverse transcription) showed no amplicon, which confirmed the absence of genomic DNA. The PCR efficiency (*E*) and correlation coefficients (*R*^2^) were determined based on the slopes of the standard curves generated from the *C*q values of serial dilutions of the cDNA. The *E* values of six reference genes and *IgM* ranged from 91.7% (*IgM*) to 101.1% (*18S rRNA*), and the *R*^2^ values ranged from 0.992 for *TUBA* to 0.998 for *ACTB* as shown in Table 1.

**Table 1:** Primer sequences and PCR efficiencies.

Primer Name	Sequence (5'→3')	Amplification Size (bp)	PCR Efficiency (%)	Correlation Coefficients
18S rRNA-F	GGACACGGAAAGGATTGACAG	111	101.1	0.997
18S rRNA-R	GTTCGTTATCGGAATTAACCAGAC			
ACTB-F	GACCCACACAGTGCCCATCT	140	97.7	0.998
ACTB-R	TCTCGGCTGTGGTGGTGAA			
TUBA-F	AGCCAGACGGACAGATGCC	153	92.4	0.992
TUBA-R	TTCCTGCACGCACCTCATC			
UBCE-F	GCGGACAGCTTTGGAGATGA	108	93.6	0.994
UBCE-R	CGGCAGAGAGTTAGACAAAATCG			
GAPDH-F	GATAATGGCAAACTTGTCGTCG	205	92.8	0.993
GAPDH-R	ACATTGGAGCATCGGGTGAG			
EF1A-F	GCACGCTCTGCTGGCCTTT	250	94.2	0.994
EF1A-R	GCGCTCAATCTTCCATCCC			
IgM-F	GGGAAGATGAGGAAGGAAATGA	120	91.7	0.997
IgM-R	GTTTTACCCCCCTGGTCCAT			

### 2.2. Expression Levels of Reference Genes in Different Periods of Vaccination and Infection

**Figure 2:**
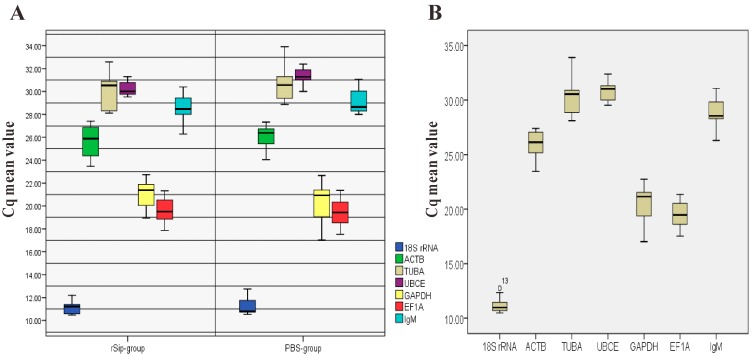
Expression levels of six candidate reference genes and *IgM* in all experimental sets presented as the *C*q mean. (**A**) *C*q values for six reference genes and *IgM* in the rSip-group and PBS-group. The line across the box is the median. The boxes indicate the 25/75 percentiles. Whisker caps indicate the minimum and maximum values; (**B**) showed *C*q values for six reference genes and *IgM* in all samples. The line across the box is the median. The boxes indicate the 25/75 percentiles. Whisker caps indicate the minimum and maximum values. The circles represent the outliers.

During vaccination and infection, the six candidate reference genes showed a wide range of expression levels in rSip-group and PBS-group, with *C*q values ranging between 10.48 and 33.92 (Table 2, Figure 2). *18S rRNA* was the highest abundannce reference gene and exhibited the highest expression levels with *C*q range between 10.48 and 12.75 throughout the different times of vaccination and infection of the rSip and PBS groups, followed by *EF1A* (17.86 to 21.36), *GAPDH* (18.48 to 22.75), *ACTB* (23.46 to 27.41), *TUBA* (28.12 to 33.92), *UBCE* (29.53 to 32.40). However, *EF1A* had lower variability at the same time in different groups with a *C*q variation between 0.04 and 0.76 when compared to *18S rRNA* (0.10 to 1.05), *ACTB* (0.08 to 2.8), *TUBA* (1.12 to 2.51), *UBCE* (0 to 2.49), and *GAPDH* (0.02 to 1.09).

**Table 2:** The *C*q values (*i.e.*, mRNA transcription levels) of six candidate reference genes and *IgM* in spleen during vaccination and infection.

Group	Time	*C*q Values (Mean ± SD)						
		*18S rRNA*	*ACTB*	*TUBA*	*UBCE*	*GAPDH*	*EF1A*	*IgM*
**rSip**	**0 ^a^**	11.20 ± 0.08	26.45 ± 0.18	30.53 ± 1.52	31.31 ± 0.77	18.95 ± 1.30	19.51 ± 0.74	29.82 ± 0.71
	**1w ^b^**	10.48 ± 0.22 *	23.46 ± 0.15 *	28.12 ± 0.38	30.22 ± 0.26	19.38 ± 1.06	17.86 ± 0.81 *	26.29 ± 0.07 *
	**2w ^b^**	11.45 ± 0.15 *	25.18 ± 0.14 *	28.50 ± 0.45	30.00 ± 0.28 *	20.71 ± 0.90 *	18.92 ± 0.70 *	27.62 ± 0.21 *
	**3w ^b^**	10.48 ± 0.10 *	23.61 ± 0.17 *	28.13 ± 0.36	29.53 ± 0.35 *	22.26 ± 0.33 *	18.79 ± 0.61 *	28.47 ± 0.18 *
	**4w ^b^**	10.70 ± 0.10 *	27.32 ± 0.18 *	30.90 ± 1.24	31.31 ± 0.38	21.53 ± 0.85 *	20.36 ± 0.35 *	29.40 ± 0.31
	**C24h ^c^**	12.20 ± 0.11 *	27.41 ± 0.33 *	32.59 ± 0.55	29.60 ± 0.21 *	22.75 ± 1.54 *	21.32 ± 0.68 *	28.40 ± 0.43 *
	**C2w ^c^**	11.31 ± 0.07	25.87 ± 0.13 *	30.87 ± 0.45	29.91 ± 1.06	21.38 ± 1.04 *	20.65 ± 1.14 *	29.05 ± 1.29
**PBS**	**0 ^a^**	11.20 ± 0.08	26.45 ± 0.18	30.53 ± 1.52	31.31 ± 0.77	18.95 ± 1.30	19.51 ± 0.74	29.82 ± 0.71
	**1w ^b^**	10.71 ± 0.08 *	25.34 ± 0.15 *	30.56 ± 2.08	31.10 ± 0.32	18.48 ± 0.75	18.62 ± 1.28 *	28.00 ± 0.12 *
	**2w ^b^**	10.54 ± 0.06 *	24.05 ± 0.04 *	29.66 ± 0.70	30.00 ± 0.23 *	19.62 ± 1.12 *	18.35 ± 0.72 *	28.30 ± 0.17 *
	**3w ^b^**	11.13 ± 0.11	26.41 ± 0.13	30.64 ± 1.07	30.96 ± 0.37	21.41 ± 0.94 *	19.43 ± 0.93	28.65 ± 0.19 *
	**4w ^b^**	10.80 ± 0.45	25.63 ± 0.52 *	29.16 ± 0.83	31.30 ± 0.74	20.93 ± 1.47 *	20.53 ± 0.66 *	29.42 ± 0.61
	**C24h ^c^**	12.75 ± 0.27 *	27.33 ± 0.46 *	33.92 ± 1.75 *	31.80 ± 0.81	22.68 ± 0.63 *	21.36 ± 1.04 *	29.08 ± 0.70
	**C2w^c^**	12.36 ± 0.82	27.06 ± 0.67	31.99 ± 1.39	32.40 ± 1.52	21.36 ± 0.76 *	20.12 ± 0.49 *	29.64 ± 1.08

“^a^” = The zero day when spleen was taken from healthy tilapia samples before vaccination; “^b^” = the different weeks after vaccination with rSip or PBS; “^c^” = the different periods after infection with *S. agalactiae*. C24h and C2w mean 24 h, 2 weeks post-challenge, respectively; “*” indicates significant (*p* < 0.05) differences compared to control (0 ^a^, before vaccination).

### 2.3. Determining the Expression Stability of Reference Genes during Vaccination and Infection

In order to identify the best reference genes for qRT-PCR data normalization during vaccination and infection in tilapia, the *C*q values of each candidate reference gene were used for expression stability comparison in the Δ*C*_t_, BestKeeper, NormFinder and GeNorm programs. We also employed RefFinder to calculate the recommended comprehensive ranking. The results indicate that the best reference genes for qRT-PCR are dependent on the different experimental sets as shown in Table 3. The top two reference genes basing on the Δ*C*_t_, BestKeeper, NormFinder and geNorm programs in spleen tissue of all groups were *EF1A/18S rRNA*, *18S rRNA/UBCE*, *EF1A/18S rRNA*, *EF1A/18S rRNA*, respectively. The best three reference genes were *EF1A*, *18S* *rRNA* and *ACTB* based on the overall final ranking of RefFinder method (Table 3). With regard to expression stability of candidate reference genes in different treatment groups, *EF1A*, *18S* *rRNA* and *ACTB* were also found to be the three most stable reference genes in spleen tissue of rSip-group, followed by *TUBA*, *UBCE* and *GAPDH*, respectively (Table 3). The recommended comprehensive ranking of stability was determined as *18S rRNA* > *UBCE* >*ACTB* > *EF1A* > *TUBA* > *GAPDH* in spleen tissue of PBS-group (Table 3). Moreover, our results show that *GAPDH* is the most unstable gene under different experimental sets.

**Table 3:** Expression stability ranking of six candidate reference genes during vaccination and infection in spleen of different experimental sets.

Group	Rank	Method				
		Comparative Δ*C*_t_ (Average of SD)	BestKeeper (SD (±CP))	NormFinder (Stability Value)	geNorm (Stability Value M)	RefFinder (Overall Final Ranking)
**All groups**	**1**	*EF1A* (0.98)	*18S* *rRNA* (0.56)	*EF1A* (0.34)	*EF1A*/*18S* *rRNA* (0.77)	*EF1A* (1.32)
	**2**	*18S* *rRNA* (1.01)	*UBCE* (0.76)	*18S* *rRNA* (0.43)	-	*18S* *rRNA* (1.41)
	**3**	*ACTB* (1.07)	*EF1A* (0.91)	*ACTB* (0.64)	*ACTB* (0.84)	*ACTB* (3.22)
	**4**	*TUBA* (1.26)	*ACTB* (1.10)	*TUBA* (0.99)	*TUBA* (0.95)	*UBCE* (3.98)
	**5**	*UBCE* (1.29)	*GAPDH* (1.20)	*UBCE* (1.04)	*UBCE* (1.04)	*TUBA* (4.43)
	**6**	*GAPDH* (1.47)	*TUBA* (1.23)	*GAPDH* (1.30)	*GAPDH* (1.18)	*GAPDH* (5.73)
**rSip group**	**1**	*EF1A* (1.01)	*18S* *rRNA* (0.48)	*EF1A* (0.35)	*ACTB*/*TUBA* (0.70)	*EF1A* (1.73)
	**2**	*18S* *rRNA* (1.20)	*UBCE* (0.60)	*18S* *rRNA* (0.66)	-	*18S* *rRNA* (2.00)
	**3**	*ACTB* (1.23)	*EF1A* (0.98)	*ACTB* (0.85)	*EF1A* (0.74)	*ACTB* (2.59)
	**4**	*TUBA* (1.26)	*GAPDH* (1.13)	*TUBA* (0.93)	*18S* *rRNA* (0.97)	*TUBA* (3.13)
	**5**	*UBCE* (1.54)	*ACTB* (1.31)	*UBCE* (1.29)	*UBCE* (1.15)	*UBCE* (3.98)
	**6**	*GAPDH* (1.63)	*TUBA* (1.46)	*GAPDH* (1.42)	*GAPDH* (1.31)	*GAPDH* (5.42)
**PBS group**	**1**	*18S* *rRNA* (0.74)	*UBCE* (0.50)	*18S* *rRNA* (0.08)	*18S* *rRNA*/*UBCE* (0.51)	*18S* *rRNA* (1.19)
	**2**	*ACTB* (0.84)	*18S* *rRNA* (0.69)	*ACTB* (0.47)	-	*UBCE* (2.00)
	**3**	*EF1A* (0.87)	*EF1A* (0.83)	*EF1A* (0.52)	*ACTB* (0.57)	*ACTB* (2.63)
	**4**	*UBCE* (0.89)	*ACTB* (0.88)	*UBCE* (0.64)	*EF1A* (0.65)	*EF1A* (3.22)
	**5**	*TUBA* (1.15)	*TUBA* (1.16)	*TUBA* (0.99)	*TUBA* (0.83)	*TUBA* (5.00)
	**6**	*GAPDH* (1.17)	*GAPDH* (1.26)	*GAPDH* (1.01)	*GAPDH* (0.94)	*GAPDH* (6.00)

### 2.4. The Number of Reference Genes Required for Accurate Normalization

**Figure 3:**
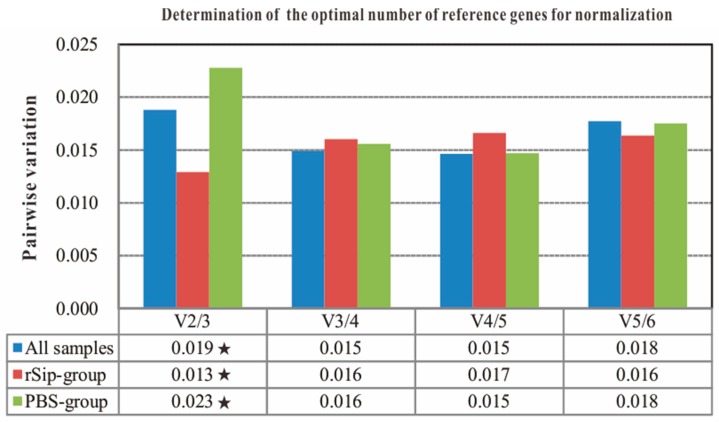
Pairwise variation analyses of candidate reference genes in different experimental sets with GeNorm programs. Pairwise variation (V) was calculated to determine the minimum number of reference genes required for accurate normalization in different experimental sets. V2/3 means the number of required reference genes is suggested two. V3/4 means the number of required reference genes is suggested three, and so on. “★” indicates the value of pairwise variation less than the recommended 0.15 for each experimental set.

To determine the number of reference genes required for optimal data normalization during vaccination and infection in spleen of different experimental sets, the pairwise variation (Vn/n + 1) of one gene with others was performed by GeNorm, which used 0.15 as a cut-off threshold, and values below this indicate that the inclusion of additional reference genes is unnecessary [25]. The results are illustrated in Figure 3. The analysis showed that no matter in which experimental sets, the V2/3 value was lower than 0.15 with 0.019, 0.013 and 0.023, respectively, suggesting that two candidate reference genes should be used together as the normalization factor for immune-related gene expression level.

### 2.5. Validation of Reference Gene Selection

**Figure 4:**
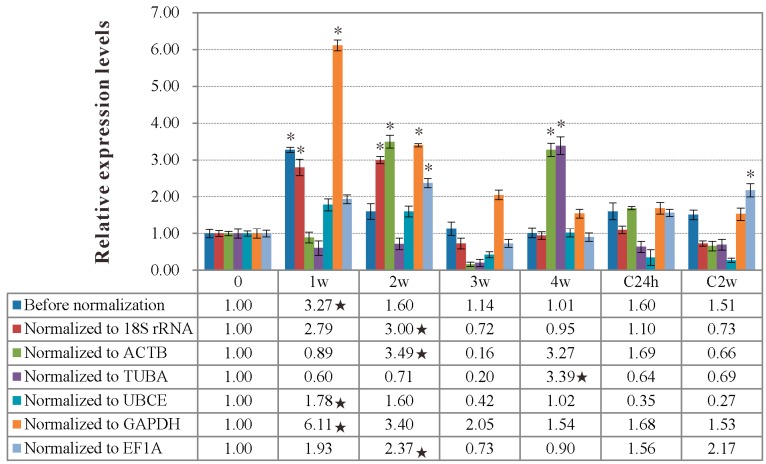
The relative expression levels of *IgM* gene in spleen of the rSip-group at the different times of vaccination and infection with *18S rRNA*, *ACTB*, *TUBA*, *UBCE*, *GAPDH* and *EF1A* as reference genes, respectively. Data was shown as Mean ± SD, compared to the PBS-group. “*” indicates significant (*p* < 0.05) differences from before vaccination (*i.e.*, 0 day); “★” indicates the highest expression levels of *IgM* when un-normalized and normalized to different reference genes during vaccination and infection.

**Figure 5:**
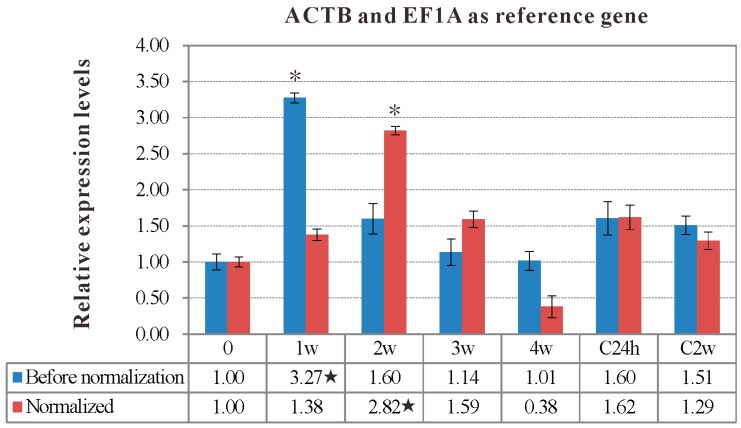
The relative expression levels of *IgM* gene in spleen of the rSip-group at the different time of vaccination and infection with *ACTB* and *EF1A* together as reference gene. Data was shown as mean ± SD, which compared to the PBS-group. “*” indicates significant (*p* < 0.05) differences from before vaccination (*i.e.*, 0 day); “★” indicates the highest expression levels of *IgM* when unnormalized or normalized to two reference genes (*ACTB* and *EF1A*) during the vaccination and infection.

The target gene *IgM* expression levels at the different time of vaccination and infection in spleen of rSip-group compared to control PBS-group were normalized to six candidate reference genes respectively. Before normalization, the *IgM* expression level was highest at 1w post-vaccination, which was consistent with the results of normalizing to *UBCE* and *GAPDH*, individually (Figure 4). The expression level of *IgM* was highest at four weeks post-vaccination when using *TUBA* as the internal control. On the contrary, the normalized expression level of *IgM* at two weeks post-vaccination was higher than at other periods of vaccination and infection when using *18S* *rRNA*, *ACTB*, *EF1A*, *ACTB* and *EF1A* together as the reference genes, respectively (Figure 5), which was consistent with the *IgM* antibody titers of the rSip-group detected by ELISA (Table 4).

**Table 4:** IgM antibody titers detected by ELISA.

Group	Time						
	0	1w	2w	3w	4w	C24h	C2w
rSip group	-	1:16	1:64	1:32	1:8	1:32	1:8
PBS group	-	-	-	-	-	1:16	-

“-” Indicates the absorbance OD_450nm_ ratios of serum sample to negative control was below 2.

## 3. Discussion

Comparative studies of the immune-related gene expression in different periods of vaccination and infection of the body provide insights into molecular mechanisms of the immune system. Likewise, accurate gene expression levels must be normalized to suitable endogenous reference genes since high variability has been noted with the qPCR method in many studies. Moreover, it is necessary and important to consider the *C*q values of candidate genes for choosing an optimal reference gene. Several studies have shown that the expression level of a reference gene may affect the results [39,40]. In this study, we selected six commonly used endogenous controls including *18S rRNA*, *ACTB*, *TUBA*, *UBCE*, *GAPDH* and *EF1A* as candidate reference genes for normalizing the expression levels of immune-related gene *IgM* and analyzed their expression stabilities during vaccination and infection in tilapia. Among them, *18S* *rRNA* had a lower average *C*q value with the range of 10.48 to 12.75 which displayed an relatively high expression level during the whole experiment in tilapia, which is similar to the findings of Yang *et al.* [15], who pointed out that *18S* *rRNA* should be selected as an internal reference gene when analyzing a high expression level target gene with the lower average *C*q value. The remaining five reference genes had a comparatively high average *C*q values, which suggested that these genes could serve as internal reference genes when analyzing a lower expression level target gene like the immune-related gene *IgM* in the present study. Therefore, it is recommended to select an optimal reference gene with a suitable *C*q value and similar expression level to target genes.

Moreover, the most important factor for selecting the suitable reference gene is the expression stability of candidate genes, regardless of tissue types, developmental periods or experiment conditions. Here, we assessed and selected reference genes with RefFinder [26], an easy and fast program that integrates the comparative Δ*C*_t_ method [22], BestKeeper [23], NormFinder [24] and GeNorm [25] to compare and rank the tested candidate reference genes, and only needed the *C*q value to make the calculation. The results indicate that neither candidate reference gene could serve as a universal normalization standard which maintained a constant expression level in spleen under various experimental stimuli. Although these candidate reference genes were considered to be classical housekeeping genes and widely used for data normalization in different species, they exhibited different variability to some extent at different periods of the same group or at the same periods of different groups in our experiment (Table 2). Examples included *EFIA*, which was the most stable reference gene in all groups, and the rSip-group ranked fourth in the PBS-group, and the top three stable reference genes of all groups and rSip-group were *EF1A*,*18S* *rRNA* and *ACTB*, while the corresponding reference genes of the PBS-group were *18S* *rRNA*, *UBCE* and *ACTB*, respectively. Interestingly, *GAPDH* is always the most unstable gene under different groups (Table 3).

Recently, increasing numbers of studies have indicated that the use of only one reference gene for normalizing qPCR data is not acceptable. It is highly recommended to use the geometric averaging of multiple reference genes for normalizing the expression of target genes of interest [25,41,42]. To determine the optimal number of reference genes throughout the experiment, we used GeNorm to calculate the pairwise variation (Vn/n + 1) of one gene with others [25]. The results indicated that no matter under which experimental set, the V2/3 value was lower than the cut-off value 0.15 with 0.019, 0.013 and 0.023 respectively, which suggested two reference genes would be sufficient for reliable normalization. Meanwhile, the combination of the reference genes *C*q value and the overall final ranking with RefFinder suggested that *EF1A* and *ACTB* were the two best reference genes used together to normalize the expression level of *IgM* during vaccination and infection in spleen of tilapia, which was well in line with the reports that two reference genes are required for accurate normalization in spleen of tilapia with *S. agalactiae* and PBS infection [15].

Immunoglobulin M (*IgM*) was commonly used as an important evaluation index of immune effectiveness, and *IgM* gene expression was significantly induced during vaccination and infection in the vaccination group in spleen of Japanese flounder (*Paralichthys olivaceus*), as well as a number of other immune relevant genes [33,34,35,38,43]. In this study, the *IgM* relative expression level was different when different candidate genes were used to normalize. When the recommended optimal gene *EF1A* and *ACTB* together were used as the reference genes, the *IgM* expression level reached the peak at two weeks post-vaccination, while the peaks of *IgM* expression level were at one week post-vaccination with no normalization, or normalization with the most unstable gene, *GAPDH*. Consistent with this, *IgM* antibody titers detected by ELISA was also highest at two weeks post-vaccination. The variation trend of *IgM* titers in serum could effectively reflect the variation of *IgM* expression level and the potency of rSip injection to elicit fish immune response to some degree, and the two variable trends of *IgM* titers and *IgM* expression level (*EF1A* and *ACTB* together as the reference genes) were similar in practice, which indicated that the two recommended genes *EF1A* and *ACTB* were suitable as reference genes for normalizing *IgM* expression during vaccination and infection. Moreover, the reference genes identified in this study will facilitate future studies of immune-related genes expression in tilapia.

## 4. Experimental Section

### 4.1. Animals and Sample Preparation

The entire experimental procedure was approved by the Committee for the Ethics on Animal Care and Experiments at Sichuan Agricultural University. Healthy Nile tilapias with body weights of 100 ± 5.0 g were purchased from a tilapia culture farm in Chongqing municipality, China. The fish were acclimatized for two weeks at 28 °C and reared in 100-L tanks at 30 °C with aeration, and fed a commercial diet twice a day throughout the study. Fish were anaesthetized with MS222 (Sigma, Beijing, China) prior to experiments involving injection and sacrifice. Before experiments, we observed the status of these fish and randomly selected five fish for the examination of bacterial recovery from blood, liver, kidney, and spleen. No bacteria were detected from any of the examined tissues of the sampled fish. Before vaccination, spleen was taken from five healthy tilapia samples, which were marked the zero days. Fish were divided randomly into two groups (40 fish/group) and vaccinated via intraperitoneal injection with 0.15 M PBS or recombinant Sip (rSip), which was suspended in sterile 0.15 M PBS (the preparation and purification of rSip are described in [32]). After four weeks post-vaccination, fish in each group were challenged by intraperitoneal injection with *S. agalactiae* in 1.5 × 10^8^ CFU/mL (50 × LD_50_). Under aseptic conditions, serum samples and spleens were collected from five individuals at 1w, 2w, 3w and 4w post-vaccination, and 24 h, and two weeks post-challenge, respectively. From these samples, the serum was used for detecting IgM titers during the experiment with ELISA, and the spleen samples were snap frozen in liquid nitrogen and stored at −80 °C until used for extracting total RNA.

### 4.2. RNA Extraction and cDNA Synthesis

Total RNA extraction used by spleen samples was performed following the manual of RNAiso Plus Kit (TaKaRa, Dalian, China). The tissues were lysed with RNAiso Plus buffer, extracted with chloroform (Sangon, Shanghai, China), and precipitated with 2-propanol (Sangon) to obtain total RNA. The extracted RNA was digested with RNase-free DNase I (TaKaRa), verified for integrity by gel electrophoresis and estimated for purity at 230, 260 and 280 nm with a NanoVue spectrophotometer (GE healthcare, Buckinghamshire, UK). Only high-quality samples in which OD_260_/OD_280_ > 1.8 and OD_260_/OD_23__0_ > 2.0 were used for subsequent cDNA synthesis. The purified RNA was adjusted to 1.0 μg/μL with nuclease-free water (TaKaRa). The cDNA were uniformly synthesized from treated RNA using an RT Primer Mix (TaKaRa) combining oligo dT primer and random 6 mers as the primer for reverse transcription according to PrimeScript™ RT reagent Kit with gDNA Eraser (Perfect Real Time) (TaKaRa). Subsequently, performing PCR using the synthesized cDNA and the RNA without reverse transcription (RNA control) as template with gene-specific primers to confirm absence of genomic DNA.

### 4.3. Primer Design

Six commonly used reference genes including *18S* *rRNA*, *ACTB*, *TUBA*, *UBCE*, *GAPDH* and *EF1A* were examined for their potential to serve as qPCR reference genes in the study of *IgM* gene expression in tilapia during vaccination and infection. The primers for these reference genes were based on sequences published in GenBank (Available online: http://www.ncbi.nlm.nih.gov/) while *IgM* sequence was based on published paper [44] (Table 5), and designed using Primer Premier 5.0 software (Premier Biosoft International, Palo Alto, CA, USA) under default parameters. The primers for BLAST searches were performed to confirm the total genes specificity of the primer sequences, and the results showed the absence of multi-locus matching at the primer site. PCR efficiency (*E*) and correlation coefficient (*R*^2^) were determined based on the slopes of the standard curves generated using serial 10-fold dilutions of sample cDNA. The efficiency was calculated as follows: *E* (%) = (10^−1/slope^ − 1) × 100 [45]. The acceptable E value was defined as between 90% and 110% [15].

**Table 5:** Candidate reference genes and target gene used in this study.

Gene Symbol	Gene Name	Function	Accession Number/Reference
*18S rRNA*	18S ribosomal RNA	Ribosomal subunit	JF698683
*ACTB*	β-actin	Cytoskeletal protein	XM_003443127
*TUBA*	α-tubulin	Cytoskeletal protein	XM_003445344
*UBCE*	Ubiquitin-conjugating enzyme	Protein degradation	XM_003460024
*GAPDH*	Glyceraldehyde-3-phosphate dehydrogenase	Glycolytic enzyme	XM_003460024
*EF1A*	Elongation factor 1 α	Protein synthesis	AB075952
*IgM*	Immunoglobulin M	Immunoglobulin	[44]

### 4.4. Quantitative RT-PCR with SYBR Green

The candidate reference genes and target gene were amplified by qPCR from spleen tissues of tilapia during vaccination with rSip or PBS and infection with *S. agalactiae*. The qPCR was carried out with the SYBR^®^ *Premix Ex Taq*™ II (Tli RNaseH Plus) (TaKaRa) in a StepOnePlus™ Real-Time PCR System (Applied Biosystems, Foster City, CA, USA). The reaction was performed in triplicate with a total volume of 20 μL containing 2 μL cDNA (*i.e.*, reverse transcription reaction solution which have eliminated genomic DNA described in 4.2), 10 μL SYBR Premix buffer (TaKaRa), 0.4 μL ROX Reference Dye (TaKaRa), 0.8 μL forward/reverse (10 μM) and 6.4 μL nuclease-free water (TaKaRa). The reaction without the template was used as the negative control in each assay. The PCR program was 95 °C for 30 s, followed by 40 cycles comprising 95 °C for 5 s, 59 °C for 30 s, and 72 °C for 30 s. The Melting curves of the qPCR products were performed at the end of each PCR to confirm that only one product was amplified and detected. The qPCR products were examined by electrophoresis on 2% agarose gels revealed strong single bands of the expected sizes.

### 4.5. Validation of Reference Gene Selection

The target gene *IgM* was used to assess the validity of candidate reference genes. The *IgM* expression level was analyzed by normalizing to six candidate reference genes with qPCR, respectively. Relative quantification of *IgM* gene of rSip-group in different periods of vaccination and infection were conducted according to *C*q value based on 2^−ΔΔ*C*t^ method when the PBS-group used as a control. Moreover, *IgM* antibody titers detection was also performed to validate the reference gene selection with serum sample by ELISA as described previously [32].

### 4.6. Statistical Analysis

All assays were performed independently in triplicate. The *C*q values collectd from qPCR were used to analysis the stability of the candidate reference genes with the comparative Δ*C*_t_ method, BestKeeper, Norm Finder and GeNorm. The comparative Δ*C*_t_ method was used to compare relative expression of pairs of genes within each sample. BestKeeper determines the standard deviation with the user selecting the best genes based on these variables. NormFinder ranks the stability of each candidate gene independently. The GeNorm algorithm calculates an expression stability value (M) for each gene and then performs a pair-wise comparison (Vn/Vn + 1) of this gene with others. Finally, we compared and performed the overall final ranking of the candidate genes based on the web-based analysis tool, RefFinder. All other multiple comparisons were performed with SPSS19.0 and the statistical significance of difference between mean values was determined by analysis of variance (ANOVA), and significance was defined as *p* < 0.05.

## 5. Conclusions

In conclusion, it is necessary and important to select stable reference genes for normalizing the interest gene expression level. However, assessment and validation of the expressing stability of candidate genes are the most critical steps for screening the optimal reference genes. In this study, we selected and validated six widely used endogenous genes (*i.e.*, *18S* *rRNA*, *ACTB*, *TUBA*, *UBCE*, *GAPDH* and *EF1A*) in the studies of gene expression and mRNA transcription level with the qPCR method, to find that these reference genes all exhibited different variabilities to some extent during the different periods of vaccination and infection in tilapia. Among them, *18S* *rRNA* had the most steady expression level but with a high abundance, while *EF1A* and *ACTB* with a suitable Cq value were the two optimal reference gene for normalizing the immune-related gene expression. However, the reference genes stability still must be determined on a case-by-case basis in tilapia studies; certain genes may be preferred for normalization in experiments involving different treatments. Last but not least, our results suggest that two reference genes, *EF1A* and *ACTB*, are recommended and sufficient for reliable normalization of immune-related genes of tilapia during vaccination and infection.
